# Simple One Pot Preparation of Chemical Hydrogels from Cellulose Dissolved in Cold LiOH/Urea

**DOI:** 10.3390/polym12020373

**Published:** 2020-02-07

**Authors:** Jiayi Yang, Bruno Medronho, Björn Lindman, Magnus Norgren

**Affiliations:** 1 FSCN, Surface and Colloid Engineering, Mid Sweden University, SE-851 70 Sundsvall, Sweden; jiayi.yang@miun.se (J.Y.); bfmedronho@ualg.pt (B.M.); bjorn.lindman@fkem1.lu.se (B.L.); 2 MED—Mediterranean Institute for Agriculture, Environment and Development, Faculty of Sciences and Technology, Campus de Gambelas, Ed. 8, University of Algarve, 8005-139 Faro, Portugal; 3 Physical Chemistry, University of Lund, SE-221 00 Lund, Sweden; 4 Chemistry Department, University of Coimbra, 3004-504 Coimbra, Portugal

**Keywords:** LiOH/urea, Michael addition, methylenebisacrylamide, cellulose hydrogel

## Abstract

In this work, non-derivatized cellulose pulp was dissolved in a cold alkali solution (LiOH/urea) and chemically cross-linked with methylenebisacrylamide (MBA) to form a robust hydrogel with superior water absorption properties. Different cellulose concentrations (i.e., 2, 3 and 4 wt%) and MBA/glucose molar ratios (i.e., 0.26, 0.53 and 1.05) were tested. The cellulose hydrogel cured at 60 °C for 30 min, with a MBA/glucose molar ratio of 1.05, exhibited the highest water swelling capacity absorbing ca. 220 g H_2_O/g dry hydrogel. Moreover, the data suggest that the cross-linking occurs via a basic Michael addition mechanism. This innovative procedure based on the direct dissolution of unmodified cellulose in LiOH/urea followed by MBA cross-linking provides a simple and fast approach to prepare chemically cross-linked non-derivatized high-molecular-weight cellulose hydrogels with superior water uptake capacity.

## 1. Introduction

Increasing environmental awareness and concern have drawn considerable attention towards using the available commodities in sustainable and environmental friendly ways. In this respect, cellulose occupies a prominent position as the most abundant biopolymer on earth. Cellulose finds a huge variety of applications ranging from paper, boards, textiles and nonwoven fabrics to roles as a reinforcing agent, thickener, emulsifier and stabilizer in different food, pharmaceutical and biomedical applications [1,2]. Cellulose is already one of the most interesting and promising candidates to compete with and eventually replace petroleum-based resources for both daily life and industrial products.

Among the vast list of possible applications, cellulose-based hydrogels have recently attracted great attention due to their numerous advantages, such as hydrophilicity, renewability, biodegradability, biocompatibility, low cost and non-toxicity [3,4,5]. Hydrogels are often defined as cross-linked polymer networks capable of absorbing significant amounts of fluids while maintaining their tridimensional structural integrity. Due to superior water absorbency, these systems can be used in many different areas such as in personal healthcare [6], agriculture [7], biomedicine [8], construction [9] and among others [10].

Biopolymer-based hydrogels have also been extensively used in the food and pharmaceutical industries [11] in addition to state-of-the-art and promising applications in tissue engineering [12,13,14], drug delivery [15,16] and microfluidics [17,18]. These systems can be doped with different drugs, nanoparticles or other molecules of interest which may not only tune their viscoelastic features but also enhance their potentials for applications, affect their release profiles and even allow specific processing, such as their injection [19]. In this respect, the mechanical and flow properties cannot be neglected when judging the feasibility of a hydrogel for a specific biological application. For instance, suitable flow properties allow hydrogels to be exciting candidates as injectable therapeutic delivery carriers if they are capable of shear-thinning during application at a proper shear stress; rapid self-healing; and adopting a solid-like behavior once the stress is removed [20].

The general processing of cellulose, including the hydrogel formation, is challenging, since the biopolymer does not dissolve in water or in common organic solvents. Cellulose dissolution requires the disruption of the relatively extended inter- and intramolecular hydrogen bond network within its structure, as well as the van der Waals and hydrophobic interactions among the less polar faces of the anhydroglucose units [21,22,23,24].

It is thus not surprising that for cellulose-based hydrogels the majority of studies have mainly focused on systems involving cellulose derivatives that can be easily dissolved in aqueous media [3,5,25,26]. Some studies have focused on physical hydrogels from several cellulose derivatives [27,28], while chemical cross-linking is more commonly used [3]. In this respect, chemically crosslinked cellulose-based hydrogels have been prepared using different methodologies, such as esterification [29], and radical and graft polymerization [30,31]. Methods involving free-radical initiation using an initiator compound or a high energy irradiation source, such as microwaves, *γ*-rays or glow discharge by electrolysis plasma, have also been suggested [29,32,33,34,35,36,37,38,39]. Most of these processes involve time-consuming procedures requiring large quantities of chemicals, which make the post-treatment challenging. Consequently, these are generally expensive and poorly efficient strategies [32].

Chemical cross-linking overcomes the problem of poor robustness of physical hydrogels and very often N,N’-methylene-bis-acrylamide (MBA) is used as a cross-linking agent owing to its two highly reactive carbon‑carbon double bonds [40,41]. This cross-linking agent can react with different groups, such as -NH_2_, -OH and -COOH, forming a three-dimensional network [42,43]. MBA has also been successfully used to reinforce hybrid cellulose-based systems, such as carboxymethyl cellulose (CMC)-polyacrylic acid [44], CMC-alginate [45], CMC-montmorillonite superabsorbent hydrogels [46], a semi-interpenetrating network of poly(NIPAm) and cellulose nanowhiskers [47], CMC-CMC-g-poly(acrylic acid-co-acrylamide) [48], bacterial cellulose-acrylamide [49], cotton with acrylic acid and acrylamide [40], and others [50]. The MBA has also been shown to be a safe cross-linker in biomedical applications, since its toxicity can be considerably reduced when the hybridized carbon atoms are converted from sp2 to sp3 during the cross-linking process [51,52].

As previously mentioned, the poor solubility of unmodified cellulose has been limiting its direct application in the formation of hydrogels. Nevertheless, during the last few decades, several solvents have been successfully developed, and among them the NaOH/urea solvent system is particularly relevant [53]. It is a fairly inexpensive and low-toxic system that has been employed in the preparation of hydrogels and aerogels from non-derivatized cellulose solutions [10,53]. However, the hydrogels formed usually present poor mechanical properties, low transparency and low water absorption [54,55]. In this respect, Yuan et al. has presented a pioneering work where robust hydrogels were prepared from solutions of unmodified cellulose in NaOH/urea and further crosslinked with MBA without the need for any initiator or high energy inputs [56]. Following that work, Geng has recently shown the possibility of developing unmodified cellulose-based hydrogels and aerogels with various degrees of transparency and swelling ability, from NaOH/urea at room temperature using MBA [4,43].

Due to the high demand of simple, efficient, inexpensive and “green” technologies to produce cellulose hydrogels, in this work we revisit a cold alkali-based system based on the LiOH/urea mixture. This is an innovative approach, since to our knowledge, this direct cellulose solvent has not been used before for the formation of unmodified cellulose hydrogels chemically crosslinked with MBA. This solvent is more efficient than the previously reported NaOH/urea system and can dissolve cellulose of higher molecular weight, such as pulp [57]. The hydrogels formed under different conditions were fully characterized regarding their respective rheology, structural features and swelling capacities.

## 2. Materials and Methods

### 2.1. Material

The cellulose used was a commercial sulphite dissolving pulp (henceforth simply designated as cellulose), with a weight average molecular weight (*M_w_*) of 3.2 × 10^5^ g mol^−1^ and a polydispersity index of 10, provided by Domsjö Fabriker Aditya Birla (Örnsköldsvik, Sweden). Analytical grade crosslinking reagent, methylenebisacrylamide (MBA), was purchased from Sigma-Aldrich (Merck, Stockholm, Sweden). The other chemicals: lithium hydroxide (LiOH), urea and silicone oil, were of analytical grade and supplied by VWR Prolabo^®^ chemicals (Avantor, Stockholm, Sweden). Deionized water was used in sample preparation.

### 2.2. Dissolution of Cellulose

According to our previously published methods [58], solutions with 2, 3 and 4 wt% cellulose were prepared separately via dissolving the corresponding amounts of cellulose fiber in aqueous solvent containing LiOH/urea/water (4.6:15:80.4 w/w); 2, 3 and 4 g of cellulose (oven dried, O.D.) were dispersed with extensive stirring in 98, 97 and 96 g of thawed LiOH/urea solvent, respectively [58,59]. Cellulose solutions were kept at −35 °C until they were completely frozen, and were then thawed at room temperature and stirred at 1300 rpm for 2 min. This freezing/thawing/stirring cycle was repeated two more times until cellulose was fully dissolved. The 2, 3 and 4 wt% transparent cellulose solutions were obtained after removing the air bubbles by centrifuging the samples at 8000 rpm and 0 °C for 10 min (Beckman Culter, Avanti J-25 with JLA-16.250 Fixed Angle Rotor, Indianapolis, IN, USA).

### 2.3. Preparation of Chemically Crosslinked Cellulose Hydrogels

Cellulose hydrogels were prepared by directly mixing the 2 wt% cellulose solutions with MBA at different MBA/glucose molar ratios (Table 1). The desired amount of MBA was simply added to cellulose solution under 1300 rpm agitation, and blended at 23 °C for 2 h. The mixture was then transferred to a Petri dish and allowed to cure either at 60 °C for 30 min or at 23 °C for 12 h. The MBA/glucose molar ratios, experimental conditions and the nomenclature for coding the different samples, are listed in Table 1.

### 2.4. Characterization of Cellulose Hydrogels

#### 2.4.1. Swelling Degree

The swelling degree (SD) was applied for evaluating of the water adsorption capacity of the hydrogel. Briefly, the cured hydrogels were immersed in excess deionized water to guarantee the release of all solvent components (i.e., LiOH and urea) and unreacted MBA to the aqueous bulk. During this process, fresh deionized water was changed every 12 h until the pH reached 7. The SD was estimated from the following equation:

(1) $$SD\;\%=\frac{W_{2}-W_{1}}{W_{1}}\times 100$$

where $W_{1}$ is the weight of the lyophilized hydrogel and $W_{2}$ is the weight of the swollen hydrogel. Note that lyophilized hydrogel was taken as the reference state that corresponds to a low density material with high surface area and porosity which may be suitable for many advanced applications [60]. These lightweight systems are often coined as “aerogels” but are better described as “foams” due to their high levels of macroporosity [61,62,63]. The weight of the swollen hydrogel was measured after removing the excess water by gently tapping the surface with a dry filter paper.

#### 2.4.2. Rheometry

The rheological measurements were performed using a rotational rheometer (MCR 501; Anton Paar Physica, Ostfildern, Germany) with a plate-plate stainless steel geometry (50 mm diameter and 1 mm gap). Sample evaporation during measurements was minimized using a small amount of low viscosity silicone oil, which was placed on the periphery of samples (hydrogel-air interface) together with an evaporation blocker. Isothermal dynamic frequency and amplitude sweep tests were performed to determine the linear viscoelastic properties. The frequency and strain amplitude used within the linear viscoelastic regime were 1 Hz and 10%, respectively, in all the dynamic tests performed. Different temperature ramps were also performed to evaluate the thermal effect on gelation.

#### 2.4.3. Fourier Transform Infrared Spectroscopy (FT-IR)

The systems were also characterized using Fourier transform infrared spectroscopy (FT-IR) (Nicolet 6700; Thermo Scientific, Waltham, MA, USA), and all the samples were examined using a 4 cm^−1^ resolution and 64 scans in the range of 4000–400 cm^−1^. Background spectra were collected before every analysis. All the hydrogels were freeze-dried before analysis.

#### 2.4.4. Raman Spectroscopy

Non-polarized Raman spectra of the lyophilized hydrogels was recorded with a Horiba Xplora™ plus equipped with a 532 nm laser and Olympus BX40 confocal microscope (Horiba Xplora™ plus; Horiba, Les Ulis, France). The entrance slit width was fixed to 100 μm. The laser power on the sample was adjusted by means of density filter to avoid uncontrolled thermal effects. The spectral resolution was about 1.0 cm^−1^.

#### 2.4.5. X-Ray Diffraction (XRD)

The diffraction patterns were obtained using an X-ray diffractometer (XRD) (D phaser; Bruker, Billerica, MA, USA) with a Cu Kα radiation of 1.54 Å at 30 kV and 10 mA, and the patterns were recorded in the 2θ region from 5° to 45° at a scanning rate of 0.01° s^−1^.

#### 2.4.6. Electronic Microscopy

The morphologies of the dried cellulose hydrogels were characterized by field emission scanning electron microscopy (FE-SEM) (TESCAN MAIA 3; Oxford instrument, Abingdon, UK) at an accelerating voltage of 15 kV with a 5 nm iridium coating (Q150 T ES; Quorum Technologies, Lewes, UK).

## 3. Results and Discussion

### 3.1. Rheological Analysis of Cellulose Solution and Cellulose/MBA

Apart from the induced gelation by addition of a certain cross-linking agent, cellulose solutions are often metastable, and gelation tends to occur with temperature, pH, time or changing solvent concentration. In particular, it is well known that the stability of cellulose solutions in aqueous alkali is strongly temperature dependent. The gelation time has been found to decrease exponentially with increasing temperature [64,65]. Nevertheless, the reason for such gelation is still not fully clear [21,65,66,67]. Recently it has been suggested that the observed gelation is due to the precipitation of cellulose where the crystallites would then act as bridging points, competing for the same cellulose molecules, and thus acting as effective cross-linkers to form the 3D gel network [65]. Additionally, Isobe et al. have suggested that upon heating a cellulose solution, gelation is triggered by the hydrophobic stacking of monomolecular sheets, followed by their mutual association via hydrogen bonding [68].

**Figure 1:**
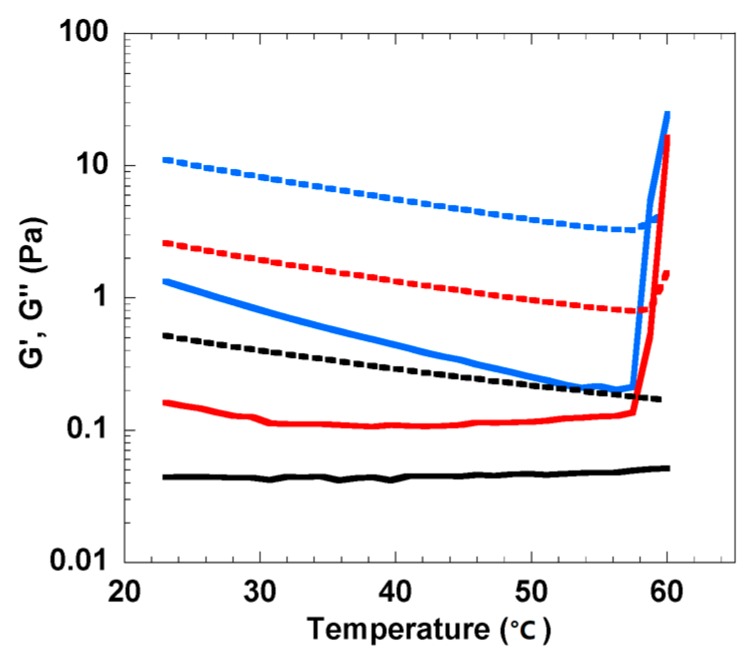
Storage modulus, G’, (solid lines) and loss modulus, G’’, (dashed lines) as functions of temperature for 2 wt% (black), 3 wt% (red) and 4 wt% (blue) cellulose solutions. The temperature ramp was performed with a heating rate of 1 °C/min at a constant frequency of 1 Hz and strain amplitude of 10%.

Based on this previous knowledge, the first step of this work consisted of selecting a proper cellulose concentration where thermal gelation was minimized. By doing so, the cellulose concentration effect on gelation could be excluded from the cross-linking agent effect. In Figure 1, temperature ramps of fresh 2, 3 and 4 wt% cellulose solutions, dissolved in LiOH/urea, are shown from 23 to 60 °C. Initially all samples display a liquid-like behavior with the loss modulus, G’’, being higher than the storage modulus, G’. The normal thermal effect is also observable with both moduli decreasing as the temperature increases. However, for the 3 and 4 wt% cellulose solutions, the moduli were observed to crossover (G’ = G’’) at ca. 59 °C and ca. 58 °C, respectively. This abrupt change indicates the temperature of gelation, T_g_; above it, G’ > G’’ and the cellulose solutions behave predominantly as solid-like materials. On the other hand, the 2 wt% cellulose solution presents overall lower G’ and G’’ moduli in comparison to the other samples of higher concentration over the entire temperature range studied. Although it is worth noting that the G’ slightly increases during the test, no crossover between G’ and G’’ was observed. Clearly, the 2 wt% cellulose solution did not gel when heated up to 60 °C.

**Figure 2:**
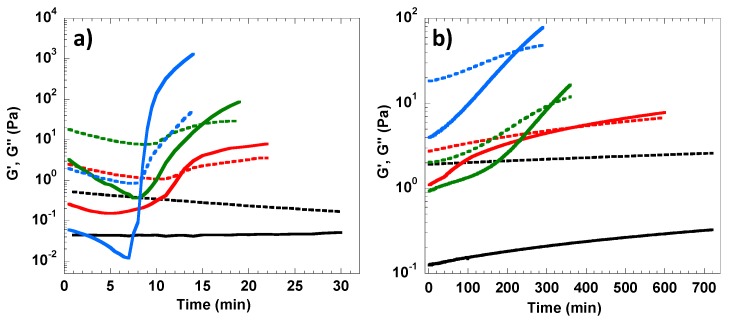
Storage modulus G’ (solid lines) and loss modulus G’’ (dashed lines) of 2 wt.% cellulose solutions with different MBA/glucose molar ratios: 0 (black), 0.26 (red), 0.53 (green) and 1.05 (blue) cured in different conditions: (**a**) temperature ramping from 23 °C to 60 °C during the first 10 min followed by 20 min at 60 °C, (**b**) cured at 23 °C for 720 min.

Since the 2 wt% cellulose solution did not gel after being heated up to 60 °C, this concentration was further selected to evaluate the effect of the MBA cross-linking agent on the gelation and hydrogel formation (see Table 1 for curing conditions and sample coding). In Figure 2a, the effect of MBA on the gelation kinetics can be observed. It is important to note that in this experiment the temperature was ramped to 60 °C during the first 10 min and then kept constant in the subsequent 20 min. In these non-equilibrium conditions only the sample with the higher MBA content (CG603) gelled during the first 10 min (i.e., T_g_ ≈ 53 °C). For lower amounts of cross-linking agent (i.e., CG601 and CG602), the sol-gel transition took place after the samples had been equilibrated at 60 °C for several minutes. In general, the higher the cross-linking amount, the faster the kinetics of gelation and the more superior the elastic properties of the hydrogel formed, particularly when compared with the systems wherein gelation was solely thermally induced without MBA (Figure 1).

The gelation kinetics were also evaluated at room temperature (i.e., 23 °C) for the different MBA contents (Figure 2b). The sample without MBA was fairly stable and did not gel at room temperature for at least, 12 h. On the other hand, when adding MBA to cellulose solutions, the gelation time was shortened as the amount of MBA increased. The gelation time dropped from 6 to 5 h, and to less than 4 h for CG231, CG232 and CG233, respectively. Overall, the data suggest that the cross-linking of cellulose with MBA can occur at room temperature but at a much slower rate; the preparation of the cross-linked cellulose hydrogels can be shortened from 6 h at 23 °C to ca. 10 min at 60 °C.

### 3.2. FTIR and Raman Analysis of Accessibility of Cellulose Hydrogel

**Figure 3:**
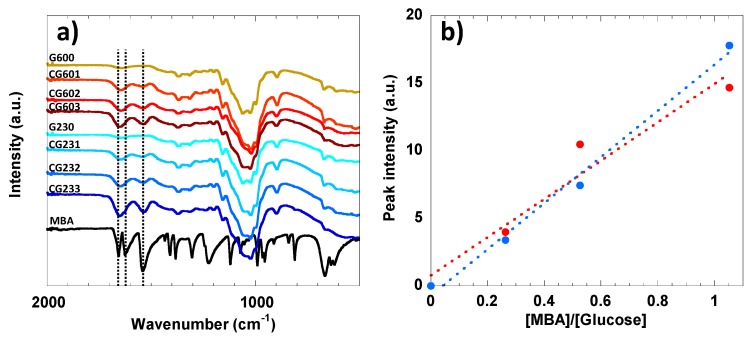
FTIR spectra of (**a**) 2 wt% cellulose samples, and (**b**) the linear regression analysis between the peak intensities of the carbonyl group at 1656 cm^−1^ and the MBA/glucose molar ratios. The vertical dashed lines at 1656 cm^−1^, 1621 cm^−1^ and 1540 cm^−1^ can be assigned to the -C=O, C=C and -NH vibration modes of MBA, respectively. The peak intensities of the -C=O group at 1656 cm^−1^ have been normalized with respect to the intensities of the common peaks at 1100 cm^−1^ and 4000 cm^−1^. The linear regressions show coefficients of determination “R” of 0.95 and 0.99 for the hydrogels cured at 60 °C (red) and 23 °C (blue), respectively.

In order to shed light into the cellulose-MBA cross-linking mechanism and hydrogel formation, a detailed FTIR analysis was performed. Figure 3a shows the FTIR spectra of the MBA and non-cross-linked cellulose references, and those of the cross-linked cellulose hydrogels cured at different conditions. In the MBA spectrum, the typical absorption bands for -C=O, C=C and -NH can be highlighted at 1656 cm^−1^, 1621 cm^−1^ and 1540 cm^−1^, respectively [69,70,71]. Once the cellulose hydrogel is formed, the C=C vibration band at 1621 cm^−1^ vanishes, which suggests that the double bonds were consumed during the crosslinking reaction. At the same time, in the hydrogel, the -C=O (1656 cm^−1^) and -NH (1540 cm^−1^) vibrational bands are still identifiable, which suggests that MBA was successfully grafted onto the cellulose chains. Regardless of the temperature, it can be noted that the intensity of the absorption band at 1656 cm^−1^ increases with the content of MBA. This can be clearly seen in Figure 3b where a linear regression analysis based on the peak intensities after normalization was conducted [72]. The data suggest that the intensity of the -C=O band in FTIR is proportional to the amount of grafted functional groups in the cellulose hydrogel, and both the reaction temperature and time significantly influence the cross-linking degree.

**Figure 4:**
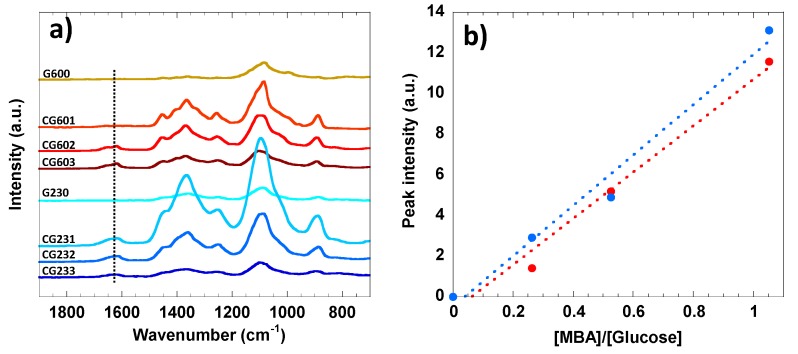
(**a**) Raman spectra between 700 cm^−1^ and 1900 cm^−1^ of 2 wt% cellulose solutions cured at 60 °C and 23 °C and with different MBA contents. The vertical dashed line at 1621 cm^−1^ can be assigned to the –C=O vibration mode from the MBA. (**b**) Linear regression analysis between the peak intensities of the carbonyl group at 1621 cm^−1^ and the MBA/glucose molar ratios. The linear regressions show coefficients of determination “R” of 0.98 for the hydrogels cured both at 60 °C for 30 min (red) and 23 °C for 12 h (blue).

In the full Raman spectra of the cellulose samples without MBA (G600 and G230), the specific bands at 1092 cm^−1^, 1362 cm^−1^ and 2887 cm^−1^, which can be assigned to asymmetric and symmetric C-O-C stretching, C-H_2_ bending and C-H stretching, respectively, could be identified (data not shown) [73,74]. The zoomed Raman spectra between 700 cm^−1^ and 1900 cm^−1^ are presented in Figure 4a. A characteristic band for cross-linked cellulose was detected at 1621 cm^−1^ and assigned to the -C=O vibration mode from the MBA [70,71]. The enhanced band intensity with MBA concentration was found to be in perfect agreement with the previously discussed FTIR data. The regression analysis of the Raman data was also conducted and compared with the FTIR data (Figure 4b). Likewise, the grafting of the -C=O onto the cellulose matrix perfectly correlates with the MBA concentration.

Raman spectroscopy has been recently used to estimate the water accessibility to the –CH_2_OH in cellulose by comparing the intensity changes of bands at 1380 cm^−1^ and 1096 cm^−1^ [75]. Agarwal et al. concluded that when soaking a polymorphous cellulose sample with D_2_O and after full OH-to-OD exchange in the cellulosic material, the increase in the intensity of the band at 1380 cm^−1^ relative to that of 1096 cm^−1^ is an indication of the non-crystalline nature of cellulose in that sample [76]. Therefore, the fraction of amorphous cellulose in the cross-linked hydrogel can be estimated from the following relation:

(2) $$amorphous\;\%=\frac{I_{1380}}{I_{1096}}*100$$

where *I*_1380_ and *I*_1096_ are the Raman spectral intensities at 1380 cm^−1^ and 1096 cm^−1^, respectively.

**Figure 5:**
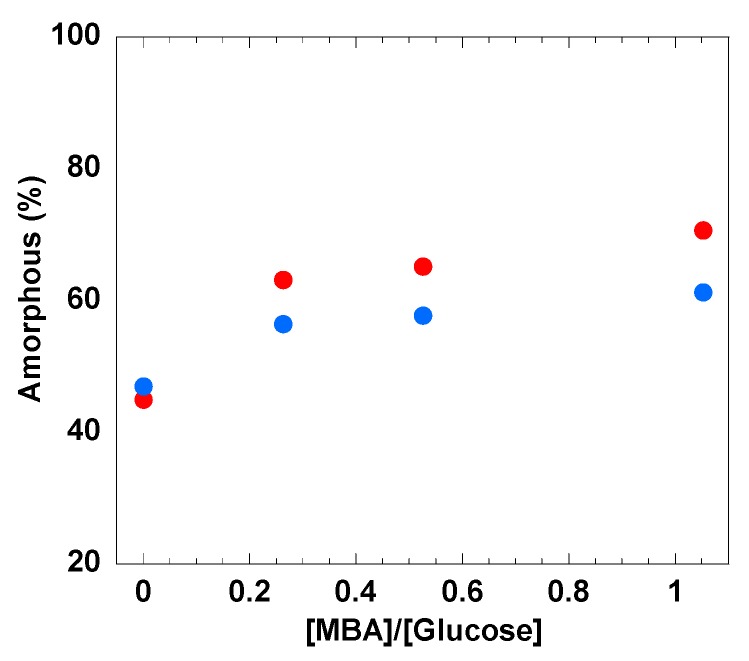
The percentages of amorphous cellulose in the hydrogels cured at 60 °C for 30 min (red symbols) and 23 °C for 12 h (blue symbols) estimated from Equation (2).

It is important to stress that despite this simple method being rather qualitative, valuable information can be still inferred from the water–hydroxyl group interactions in cellulose materials [76]. In Figure 5 the estimated numbers of amorphous regions (Equation (2)) in the synthesized cellulose hydrogels are reported for the different curing conditions. Three striking observations can be made: Firstly, it is clear that the percentage of estimated amorphous areas in the hydrogel increases with the MBA content. Most likely, the chemical cross-linking among different cellulose molecules does not enable a successful cellulose packing and formation of crystalline domains (i.e., cellulose molecules become trapped in the 3D network). Secondly, the systems without MBA show a lower amorphous cellulose content. This suggests that since no chemical bonds are formed within the hydrogel without MBA, cellulose molecular motion, physical entanglements and chain packing can be enhanced, resulting in a comparably less disordered system. Finally, the temperature effect is notable. When MBA is present, the amorphous cellulose content increases for the samples cured at higher temperature. Recently, the gelation of cellulose in alkaline aqueous solutions has been suggested as a consequence of cellulose precipitation/crystallization, where cellulose chains may participate in more than one crystallite, forming a cross-linked physical network [65].

Data strongly suggest that the MBA chemical cross-linking inhibits the system from forming more ordered crystallites, thereby resulting in a more amorphous hydrogel. The structural order is also expected to be poorer due to kinetic trapping and low possibility for molecular re-conformation (diffusion-limited aggregation).

The increment in the amount of amorphous cellulose also suggests that the accessible amount of hydroxyl groups to the water molecules in the crosslinked gels at 60 °C was increased, which could possibly indicate a better swelling capacity than for the samples prepared at 23 °C [77].

**Figure 6:**
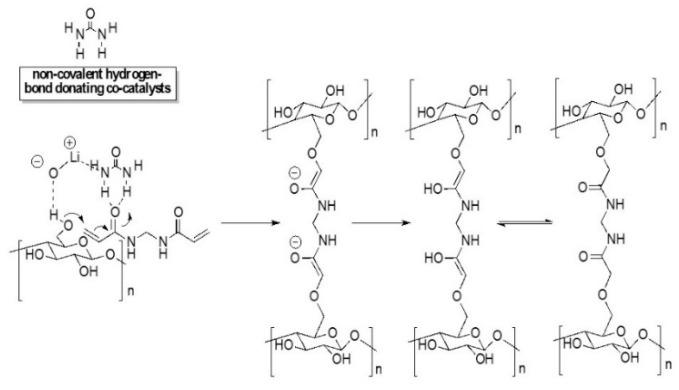
Hypothetical mechanism for hydrogel formation based on the Michael addition of cellulose to the α,β-unsaturated amide (MBA).

From a mechanistic point of view, the FTIR and Raman data support the hydrogel formation via a Michael addition-based process [78]. In Figure 6, a hypothetic reaction scheme of the MBA-cellulose cross-linking reaction is suggested. Briefly, the initial step would consist of the deprotonation of the hydroxyl group at the C6 position of the glucose anhydride catalyzed by a strong alkaline environment (i.e., LiOH aqueous solvent), forming an enolate ion as an intermediate [79]. Note that the pKa values of OH groups in the glucose units are between 12 and 12.5 [80], which means that glucose anhydride is indeed a good Michael donor in the basic Michael addition process. This nucleophile can then react with MBA, which behaves as the Michael acceptor of α,β-unsaturated ketones. The negative charge carried by the nucleophile is delocalized and saturates the alkene bond in MBA. The charge delocalization breaks the π bond of MBA and renders the oxygen with a negative charge and a double bond. In a later step, the negatively charged oxygen abstracts a proton from the water molecules. This type of mechanism has been verified in various tailored assembled macromolecular structures, ranging from linear thermoplastic to hyper-branched and cross-linked polymers. The Michael addition is one of the most useful reactions to form C-C bonds in a mild and environmentally friendly way. It can be carried out efficiently in the absence of volatile organic substitutes; with or without a catalyst in polar solvents; and either under acidic or basic conditions.

### 3.3. XRD and Morphology of the Cellulose Hydrogels

**Figure 7:**
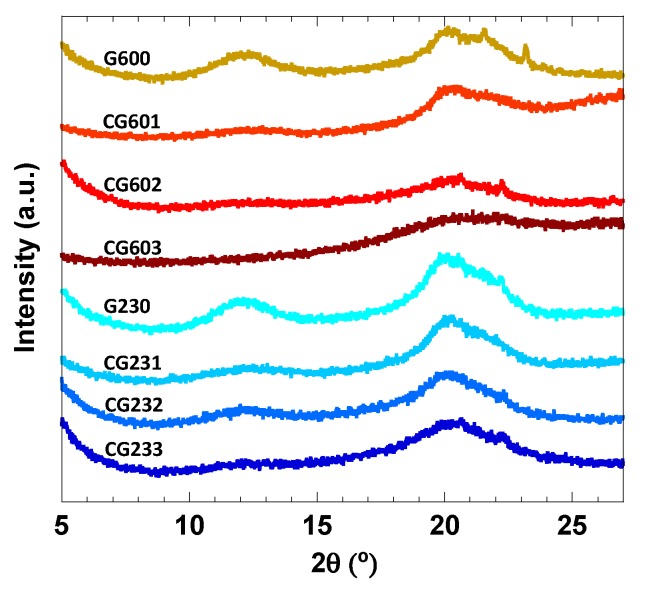
XRD spectra of the 2 wt% cellulose solutions with different concentrations of MBA and curing conditions (see table one for details).

The different cellulosic polymorphs in the hydrogels were characterized by XRD. As can be observed in Figure 7, the systems without MBA, G600 and G230 exhibited Bragg reflections at 12.4° (101) and 20.4° (002) typically assigned to cellulose II crystalline polymorph [81]. On the other hand, it is striking that once MBA is added to the system, the peak sharpness and intensity are remarkably affected. The Bragg reflections become broader and less intense which strongly suggests a decrease in crystallinity upon addition of MBA [43]. These results are thus in good agreement with the Raman data since the higher the MBA content the more amorphous the cellulose in the cross-linked hydrogels becomes. As described above, the crystalline regions of cellulose are expected to be less accessible to water molecules than the amorphous regions. Therefore, an enhanced swelling capacity of the hydrogels for the systems containing larger MBA contents can be anticipated.

**Figure 8:**
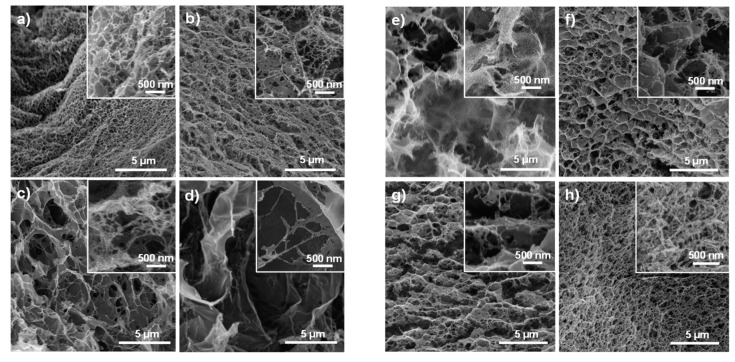
SEM images displaying the different morphological aspects of cellulose based systems cured under different conditions: (**a**–**d**) G600, CG601, CG602 and CG603, respectively; (**e**–**h**) G230, CG231, CG232 and CG 233, respectively. A higher magnification is included in the top right corner of each image.

The different systems were freeze-dried and their morphologies evaluated by SEM. As illustrated in Figure 8a, the non-crosslinked system exhibits a “foam-like” structure with an average pore size of ca. 500 nm. When MBA is added, it is striking that the pore size increases, ranging from ca. 1 μm in CG601 (Figure 8b) to several μm in the CG603 system (Figure 8d). At 60 °C (Figure 8a–d), the self-association/precipitation of cellulose molecules (physical gelation) may partially occur and most likely in a more heterogeneous fashion where the enhanced local concentration of cellulose molecules allows the physical entanglements to occur. As MBA is added, the chemical cross-linking within these areas of enhanced cellulose concentration is preferred (MBA dynamics are hindered due to local physical gelation) and expected to prevail, resulting in the formation of larger pores.

It is worth mentioning that the samples cured at 23 °C for 12 h showed very different morphological features when compared to the ones cured at 60 °C for 30 min. The sample without MBA, G230, (Figure 8e) showed larger pores after the 12 h curing, which was presumably caused by the slow and more homogeneous regeneration of cellulose molecules in the sample volume. The rheological measurements also confirmed that the sample became progressively more elastic during the 12 h. The increase in elasticity results from the increase in cellulose-cellulose links promoted by MBA addition. Since the physical gelation at lower temperature occurs at a much lower rate, the cross-linker diffusion and probability to establish chemical bonds on the entire volume increases, and thus smaller pores are expected to be formed.

### 3.4. Swelling of the Cellulose Hydrogels

**Figure 9:**
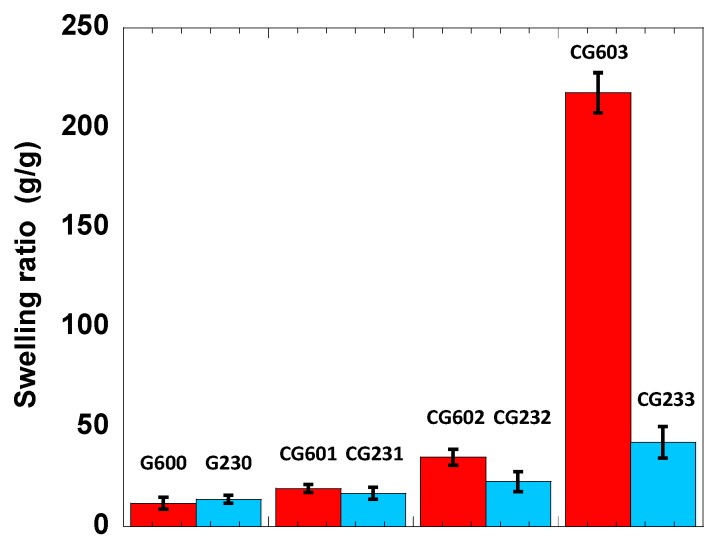
The swelling ratios of reference and crosslinked cellulose hydrogels, with CG 603 exhibiting the highest swelling ratio of 220 g/g.

The cellulose physical self-association and MBA-induced chemical crosslinks results in the formation of complex systems whose microstructures greately depend on the synthesis conditons, and thus are expected to perform differently regarding the water uptake. As described in the experimental section, the swelling capability was evaluated on the fresh hydrogels, and data are summarized in Figure 9. Once submerged in water, the hydrogel swells due to the combination of entropic and enthalpic effects of the polymer-solvent interaction and the network elasticity. The hydrophilic –OH groups from cellulose molecules and -NH groups, introduced during MBA grafting, are responsible for a favorable polymer network-water interaction triggering the solvent uptake. On the other hand, such solvent diffusion inwards the hydrogel network is counterbalanced by an elastic retractile force arising from the finite stretching-expansion of the cellulose chains and 3D network [82].

It is important to recall that the systems without MBA, i.e., G600 and G230, did not gel. However, once immersed in water, the liquid-like cellulose solution is regenerated, forming a gel, the swelling of which is independent of the previous curing conditions. Since no chemical crosslinks are formed among cellulose molecules during regeneration, both physical networks are rather similar with no significant differences in swelling capacity. On the other hand, as soon as MBA is added, two major conclusions can be drawn. Firstly, the higher the MBA concentration, the higher the water uptake. This is a general observation and can be understood by the fact that upon cross-linking, the amorphous nature of the hydrogel increases (see Figure 5). Moreover, the MBA cross-linking introduces -NH groups into the network, which can participate in hydrogen bonding with water and promote swelling of the hydrogel [83]. Both aspects favor water accessability and uptake by the cellulose-based hydrogel. The second conclusion is that when MBA is present, swelling is greatly enhanced for samples cured at higher temperatures. In this respect, the G603 system is, by far, the most efficient system with a swelling capacity of ca. 220 g/g. Nevertheless, this value is lower than that for hydrogels formed from the related NaOH/urea system, ca. 330g/g [4]. This difference is most likely related to the cellulose type used; the cellulose pulp used in this work has a much higher molecular weight than the one used in previoulsy reported NaOH/urea system. Therefore, for the same cellulose concentration, a very entangled and robust 3D network is expected for the higher molecular weight cellulose, which may hinder extensive swelling. This is also supported by a generally higher G’ (solid character) of the hydrogels formed from the LiOH/urea system in comparition to the NaOH/urea solvent.

As previously discussed, the CG603 sample has a higher number of amorphous regions in the cellulose matrix, which leads to a hydrogel with improved accessibility to water molecules. On the other hand, the CG233 sample, containing the same amount of MBA, shows lower swelling capacity than CG603. This could be explained by the fact that the systems cured at room temperaure for a longer time may render excessive grafting of the cross-linking cellulose network, which ultimatly leads to a higher rigidity of the the hydrogel, restraining the mobility of the molecular chains and ultimately causing limited swelling [84].

Overall, the swelling ratio profiles agree well with the SEM images. While the systems cured at room temperature for a longer time present a more complex and bridged network (making the hydrogel more rigid and limiting the swelling), the systems cured at high temperature display a lower degree of connectivity (larger pores), and thus can sustain a larger water uptake.

## 4. Conclusions

In this work, MBA cross-linked cellulose hydrogels with improved swelling ratios were successfully prepared, for the first time, with non-derivatized cellulose dissolved in LiOH/urea. This system can dissolve significant amounts of high-molecular-weight cellulose. The data (FTIR and Raman) suggest that grafting of MBA onto cellulose molecules occurs via a basic Michael addition mechanism. It was also verified that the addition of MBA and curing conditions have significant impacts on the amount of amorphous cellulose, the microstructure and the swelling capacity of each hydrogel. In general, the cross-linked hydrogels prepared at 23 °C rendered more crystalline regions than the ones cured at 60 °C. Both the physical self-association of cellulose molecules and the chemical cross-linking are expected to contribute to a more rigid microstructure with higher pore density and superior connectivity limiting the water uptake. On the other hand, the highest swelling ratio (ca. 220 g/g) was obtained for the system cured at 60 °C for 30 min with the higher MBA content. In this case, not only was the amorphous cellulose content the highest, but also the network elasticity (larger pores) and –NH content were favorable for water absorption. Overall, this work represents a fast and easy way of preparing non-derivatized cellulose hydrogels with tunable microstructures and significant water uptake capacities.

## Figures and Tables

**Table 1:** Molar ratios of MBA/glucose and sample nomenclature for the different curing conditions.

Reaction Conditions		n_[MBA]_/n_[Glucose]_			
		0	0.26	0.53	1.05
Temperature/°C	Time/h	Sample Code			
60	0.5	G600	CG601	CG602	CG603
23	12	G230	CG231	CG232	CG233
